# Supplementary material for: Comparative Analysis of Single-Cell RNA Sequencing Methods with and without Sample Multiplexing
Source: Int J Mol Sci. 2024 Mar 29;25(7):3828. doi: 10.3390/ijms25073828 (PMC11011421; doi:10.3390/ijms25073828)
Supplement: Supplementary file 1 [file ijms-25-03828-s001.zip › ijms-2867317-Supplementary Figures.pdf]

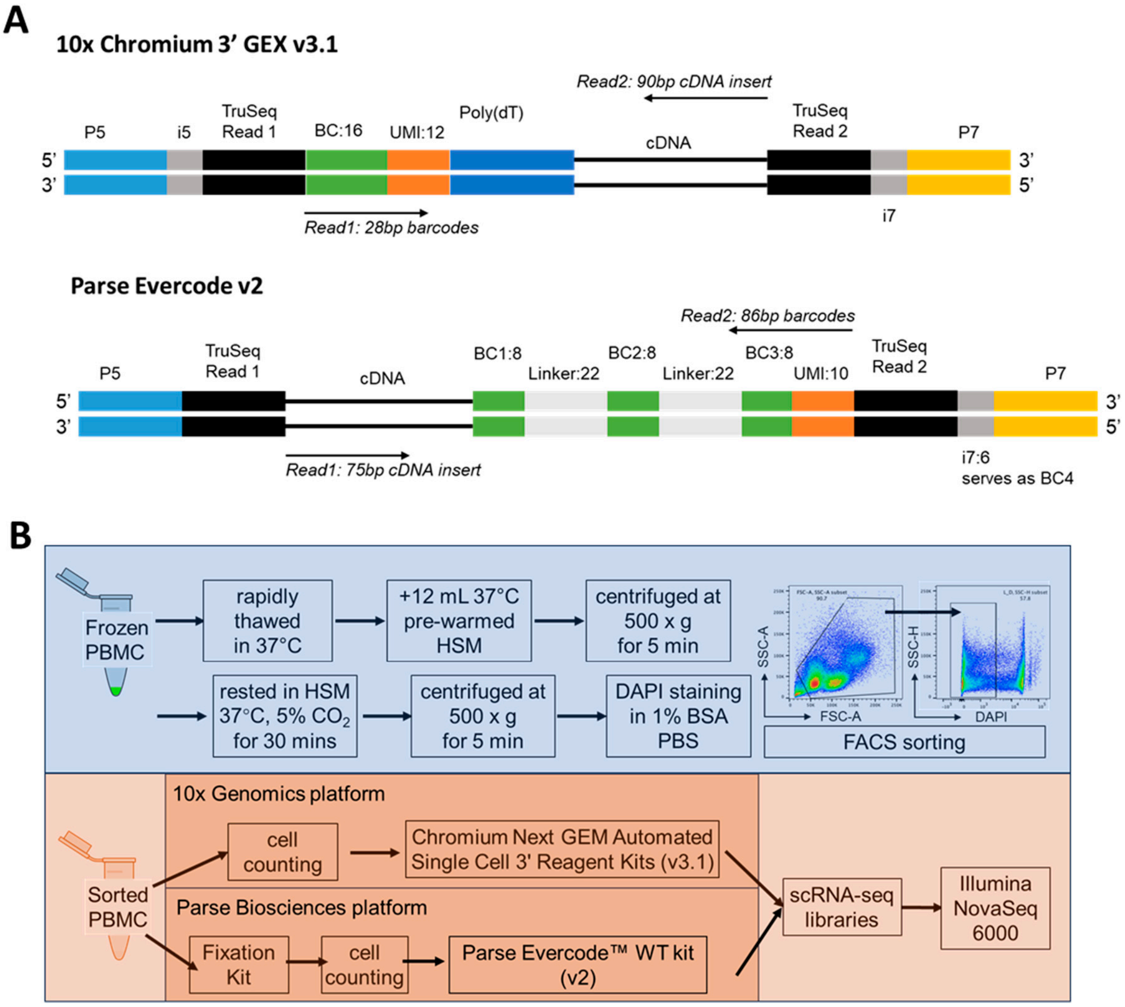

Supplementary Figure S1. Library construction. A) cDNA library structure in 10x and Parse. BC: barcode, UMI: unique molecular identifiers. **B)** Flowchart of sample preparation and library construction with 10x and Parse.

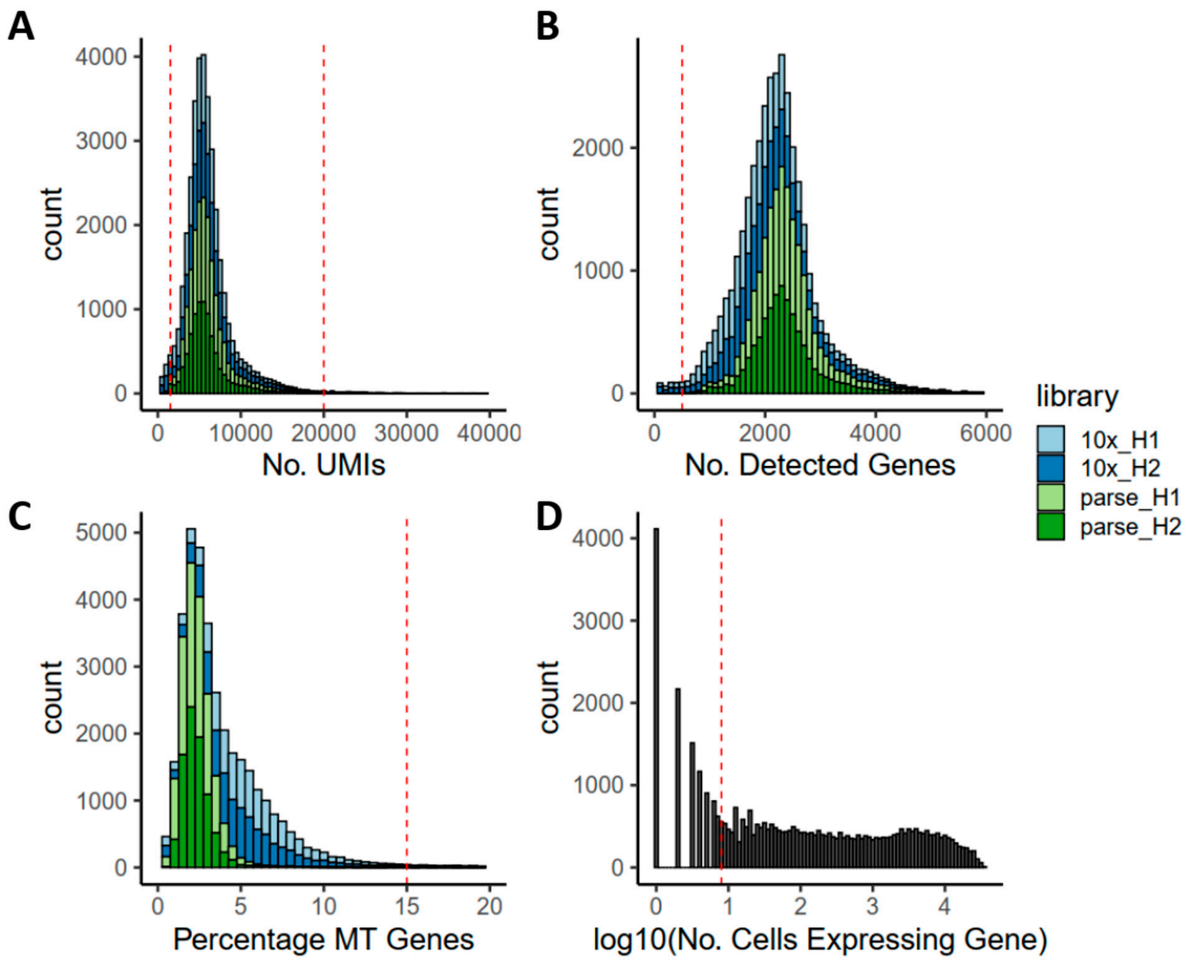

Supplementary Figure S2. Quality control. A) Distribution of UMIs detected per cell. **B)** Distribution of genes detected per cell. **C)** Distribution of percentage of UMIs mapped to mitochondrial genes per cell. **D)** Distribution of number of cells expressing the gene. Red dashed lines represent thresholds we used to filter low-quality cells and lowly-expressed genes.

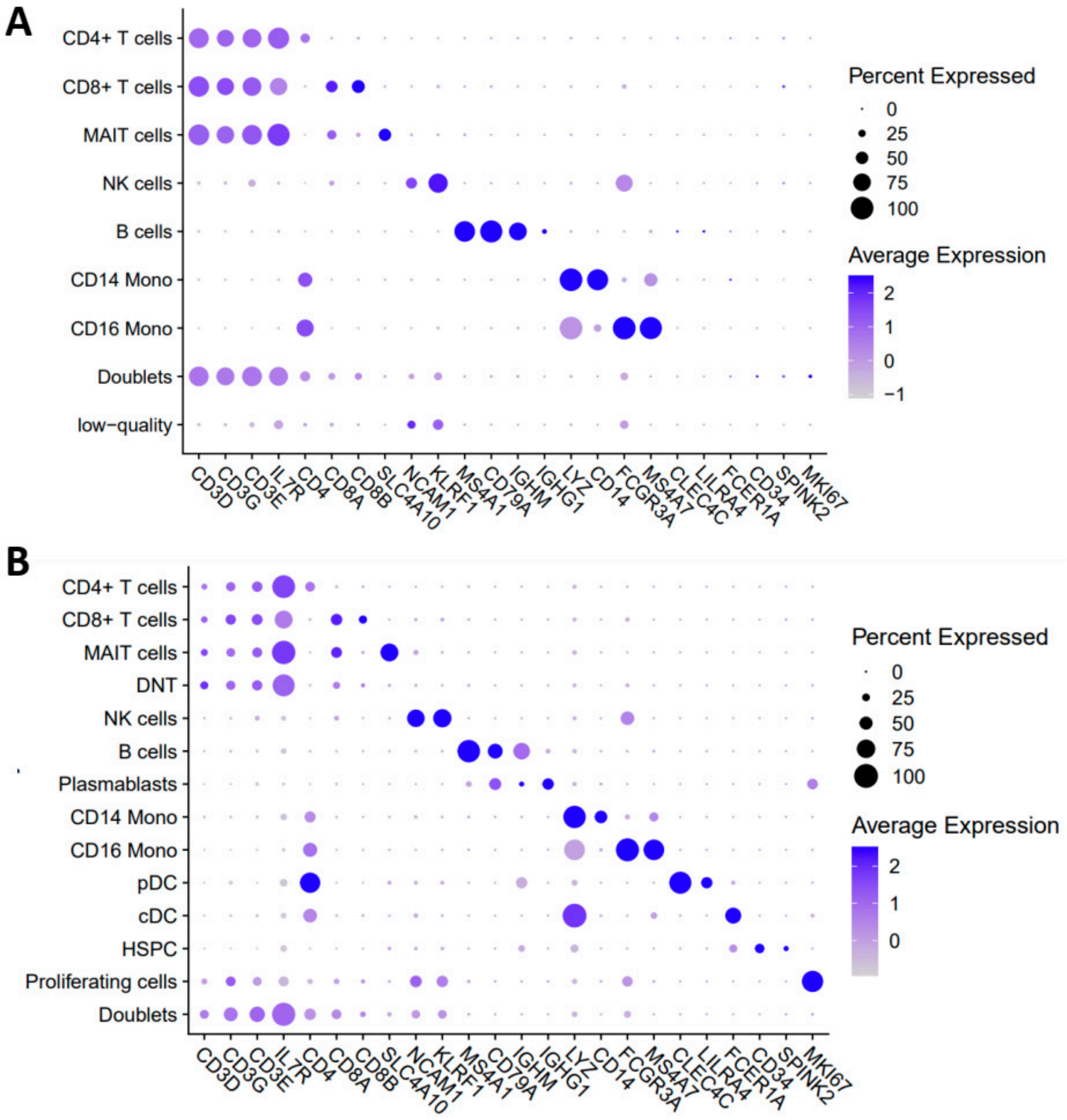

Supplementary Figure S3. Cell type annotation. A) Dotplot of average expression of cell type marker genes in 10x data. **B)** Dotplot of average expression of cell type marker genes in Parse data.

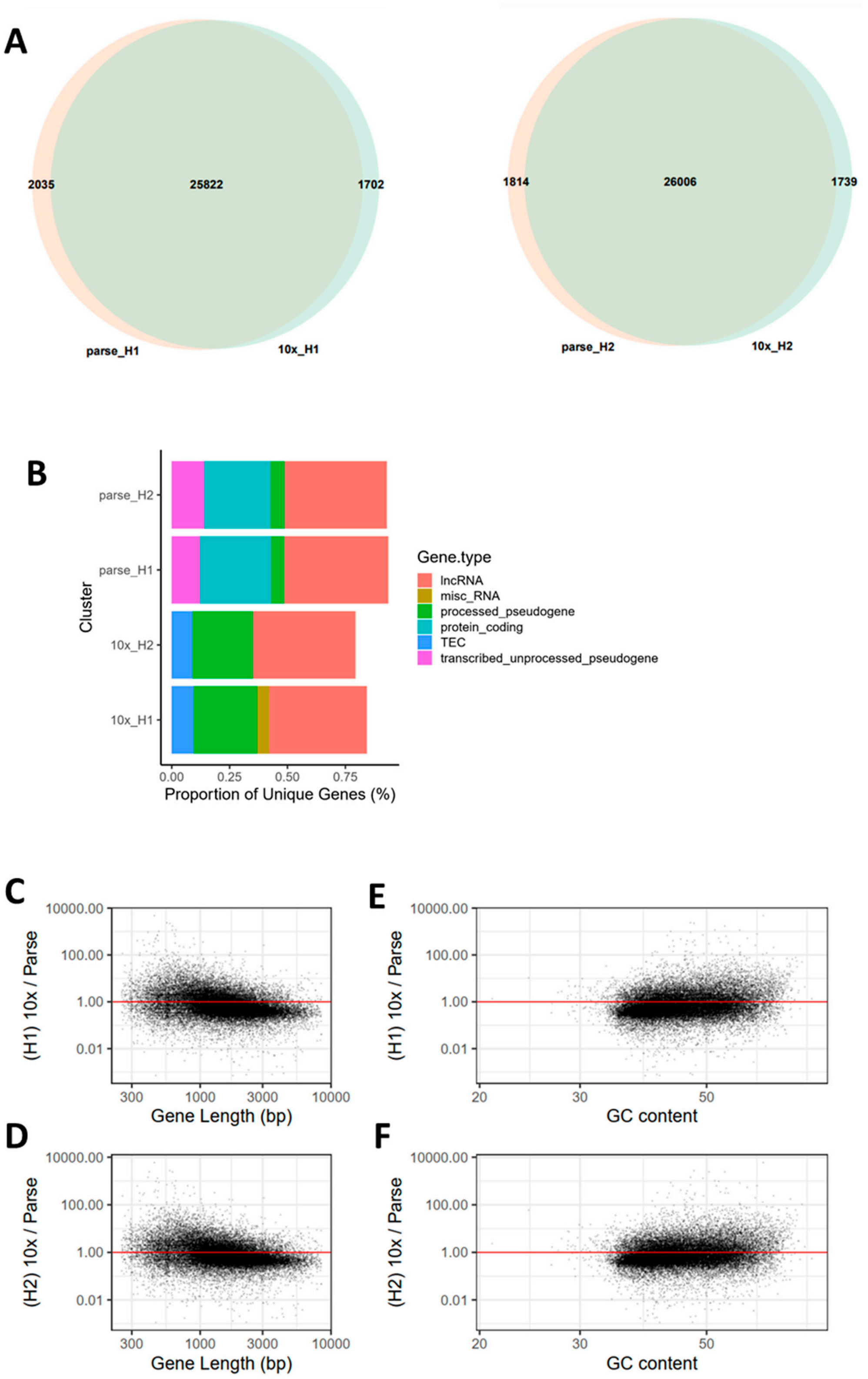
A
2035
25822
1702
parse_H1
10x_H1
1814
26006
1739
parse_H2
10x_H2
B
parse_H2
parse_H1
10x_H2
10x_H1
Cluster
0.00 0.25 0.50 0.75
Proportion of Unique Genes (%)
Gene.type
lncRNA
misc_RNA
processed_pseudogene
protein_coding
TEC
transcribed_unprocessed_pseudogene
C
(H1) 10x / Parse
Gene Length (bp)
E
GC content
D
(H2) 10x / Parse
Gene Length (bp)
F
GC content

G

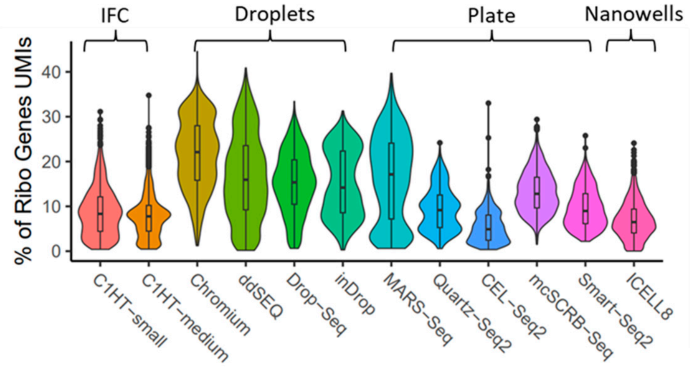

H

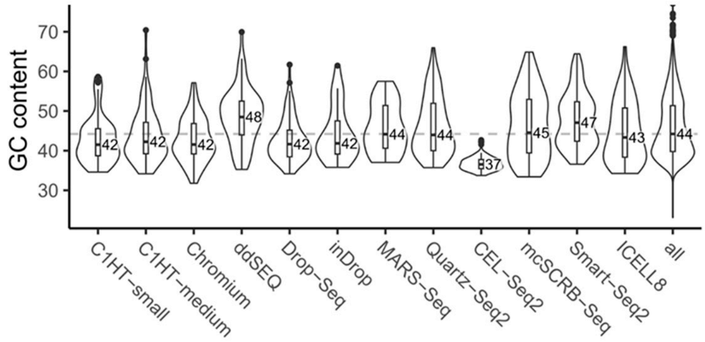

Supplementary Figure S4. Differences in gene quantification. A) Number of shared and unique genes in sample H1 (left) and H2 (right) between Parse and 10x. **B)** The abundance of gene subtypes in each method's uniquely detected genes. **C, D)** Scatterplots of gene expression ratio and gene length in sample H1 (C) and H2 (D). Each dot represents one gene that is expressed in all samples. Y axis is the ratio of average gene expression between 10x and Parse. **E, F)** Scatterplots of gene expression ratio and GC content in sample H1 (E) and H2 (F). Each dot represents one gene that is expressed in all samples. Y axis is the ratio of average gene expression between 10x and Parse. **G)** Ribosomal protein coding gene abundance in human PBMC datasets generated with 12 different scRNA-seq protocols. Cell capture technology of each protocol is indicated (IFC: integrated fluidic circuit; C1HT: Fluidigm; Chromium: v2, 10x Genomics). **H)** Distribution of gene GC content in each method's top 100 marker genes.

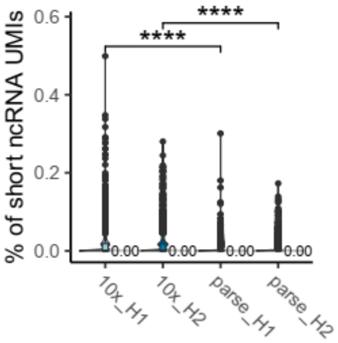

Supplementary Figure S5. Percentage of UMIs mapped to short non-coding RNAs.

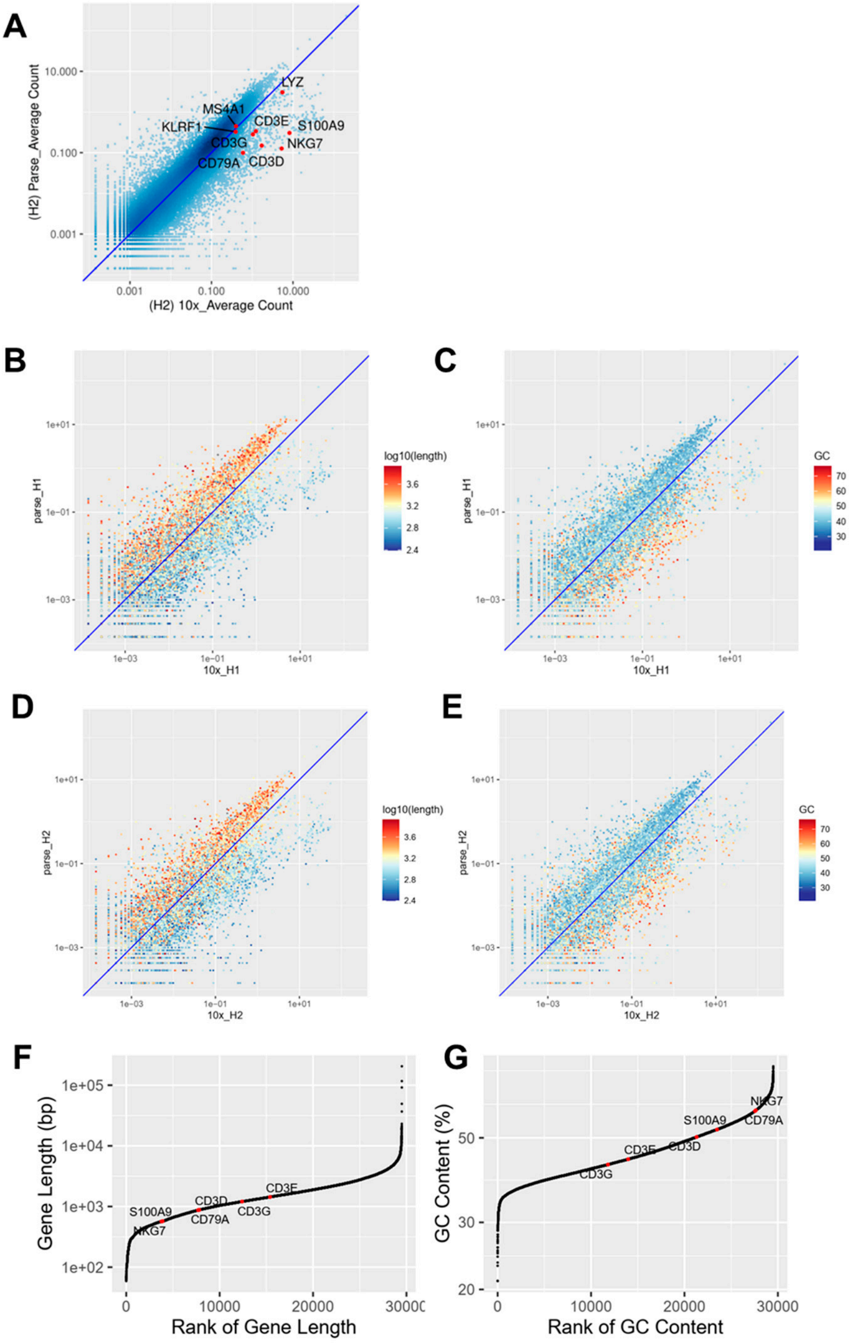

Supplementary Figure S6. Bias in gene expression quantification of cell type markers. A) Scatterplot showing gene expression correlation between two platforms in sample H2 colored by density. X-axis is the average UMI count for each gene in 10x data and Y -axis is the average UMI count for each gene in Parse data. Cell type markers are highlighted with red dots. **B, C)** Scatterplot colored by gene length and GC content in sample H1. **D, E)** Scatterplot colored by gene length and GC content in sample H2. **F)** Gene lengths of all expressed genes against corresponding ranks. **G)** GC contents of all expressed genes against corresponding ranks.
